# Predictive spatial risk model of poliovirus to aid prioritization and hasten eradication in Nigeria

**DOI:** 10.1186/1741-7015-12-92

**Published:** 2014-06-04

**Authors:** Alexander M Upfill-Brown, Hil M Lyons, Muhammad A Pate, Faisal Shuaib, Shahzad Baig, Hao Hu, Philip A Eckhoff, Guillaume Chabot-Couture

**Affiliations:** 1 Institute for Disease Modeling, Intellectual Ventures, 1555 132nd Ave NE, Bellevue, USA; 2 Duke Institute for Global Health, Duke University, Durham, USA; 3 National Polio Emergency Operations Center, Abuja, Nigeria; 4 National Primary Health Care Development Agency, Abuja, Nigeria; 5 University of Alabama at Birmingham, Birmingham, USA; 6 Kano Polio Emergency Operations Center, Kano, Nigeria

**Keywords:** Polio eradication, Spatial epidemiology, Risk modeling, Disease mapping

## Abstract

**Background:**

One of the challenges facing the Global Polio Eradication Initiative is efficiently directing limited resources, such as specially trained personnel, community outreach activities, and satellite vaccinator tracking, to the most at-risk areas to maximize the impact of interventions. A validated predictive model of wild poliovirus circulation would greatly inform prioritization efforts by accurately forecasting areas at greatest risk, thus enabling the greatest effect of program interventions.

**Methods:**

Using Nigerian acute flaccid paralysis surveillance data from 2004-2013, we developed a spatial hierarchical Poisson hurdle model fitted within a Bayesian framework to study historical polio caseload patterns and forecast future circulation of type 1 and 3 wild poliovirus within districts in Nigeria. A Bayesian temporal smoothing model was applied to address data sparsity underlying estimates of covariates at the district level.

**Results:**

We find that calculated vaccine-derived population immunity is significantly negatively associated with the probability and number of wild poliovirus case(s) within a district. Recent case information is significantly positively associated with probability of a case, but not the number of cases. We used lagged indicators and coefficients from the fitted models to forecast reported cases in the subsequent six-month periods. Over the past three years, the average predictive ability is 86 ± 2% and 85 ± 4% for wild poliovirus type 1 and 3, respectively. Interestingly, the predictive accuracy of historical transmission patterns alone is equivalent (86 ± 2% and 84 ± 4% for type 1 and 3, respectively). We calculate uncertainty in risk ranking to inform assessments of changes in rank between time periods.

**Conclusions:**

The model developed in this study successfully predicts districts at risk for future wild poliovirus cases in Nigeria. The highest predicted district risk was 12.8 WPV1 cases in 2006, while the lowest district risk was 0.001 WPV1 cases in 2013. Model results have been used to direct the allocation of many different interventions, including political and religious advocacy visits. This modeling approach could be applied to other vaccine preventable diseases for use in other control and elimination programs.

## Background

Since the commitment by the World Health Assembly to eradicate poliovirus in 1988, the disease burden has dramatically declined by more than 99% to 223 cases reported in 2012 [1,2]. Yet, in the face of growing financial and political investments, polio remains endemic in Nigeria, Pakistan, and Afghanistan and has been repeatedly exported to other previously polio-free countries—leading the 65th World Health Assembly to declare polio eradication a “programmatic emergency for global public health” in 2012 [3].

Though substantial, the resources of the Global Polio Eradication Initiative (GPEI), including vaccines, specially trained personnel, and social mobilization campaigns, are limited and must be targeted to high-risk areas within endemic countries in order to maximize impact [4]. The GPEI supports the surveillance and survey collection designed to monitor program performance in these areas [5]. However, converting these data sources into operationally useful and scientifically valid measures of future risk can be challenging.

Furthermore, before 2010, wild poliovirus (WPV) epidemics affected large portions of northern Nigeria, and 80 to 90 different districts were reporting cases within a six-month period. Thus, the need for prioritization was low, as cases were scattered across the whole of northern Nigeria and outbreak response was the focus of the problem. The lull in reported WPV cases in 2010 coupled with the small number of WPV “infected” districts scattered across northern Nigeria in 2011 suggested that the epidemic in Nigeria had become more focused; a much smaller number of districts were contributing to WPV transmission in Nigeria. Increased focus by the program was needed to anticipate which high-risk districts were at highest risk for future cases in order to prevent further transmission. Programmatically, the need to identify the districts at highest risk was intensified by a planned surge in technical and administrative capacity, supported by the World Health Organization (WHO).

Currently, one systematic method for risk assessment at the district level has been described in the literature: the WHO’s method of regional polio risk assessment, which uses a weighted linear combination of available indicators [6]. This approach is currently used by all WHO regional offices for supplementary immunization activity (SIA) campaign planning and outbreak risk assessment. As Lowther and colleagues recognize [6], this approach has limitations: these weights are based on expert opinion, not statistical modeling, and its historical predictive accuracy has yet to be demonstrated.

Previous work has demonstrated the accuracy of WPV outbreak prediction using hierarchical statistical modeling in the African region at the country/province level [7]. This model incorporated measures of connectedness between areas and population immunity and demonstrated good predictive accuracy. However, in endemic areas, operations are carried out at the district administrative level or below; at this spatial scale, underlying causal factors, such as migration, are poorly measured.

To better support modeling of spatial heterogeneity and correlation, recent work focusing on other infectious diseases has increasingly applied Bayesian spatial modeling methods [8]. These methods have been used to map infection prevalence in unmeasured areas for malaria [9,10], schistosomiasis [11-15], soil-transmitted helminths [16-18], and filariasis [19].

Only recently in infectious disease research have these methods been used to aid in forecasting disease prevalence or incidence in a future time period [20,21]. Though spatial models of infectious disease describe reported case counts, these models rarely apply zero-inflated Poisson and Poisson hurdle models to adjust for excess zeros [22]; however, these models, with and without a spatial component, have been used to analyze other ecological and public health processes [23-26]. Rarely have zero-inflated models been used to forecast future case counts.

We present a predictive spatial hierarchical Poisson hurdle model for serotype 1 and serotype 3 WPV (WPV1 and WPV3) transmission. This model is currently incorporated in district-level prioritization planning in Nigeria. Using this modeling framework, we identify the most important risk factors of the presence of WPV and the historical number of WPV cases, and also highlight areas in which these indicators are weakly informative. Due to the sparsity of the data underlying the estimates of district-level covariates, we employ a two-part methodology in which these estimates are smoothed using a temporal hierarchical model prior to their use as inputs to the spatial Poisson hurdle models. We examine this model’s historical ability to forecast districts that will report one or more WPV cases six months in the future.

## Methods

### Description of the data

**Figure 1:**
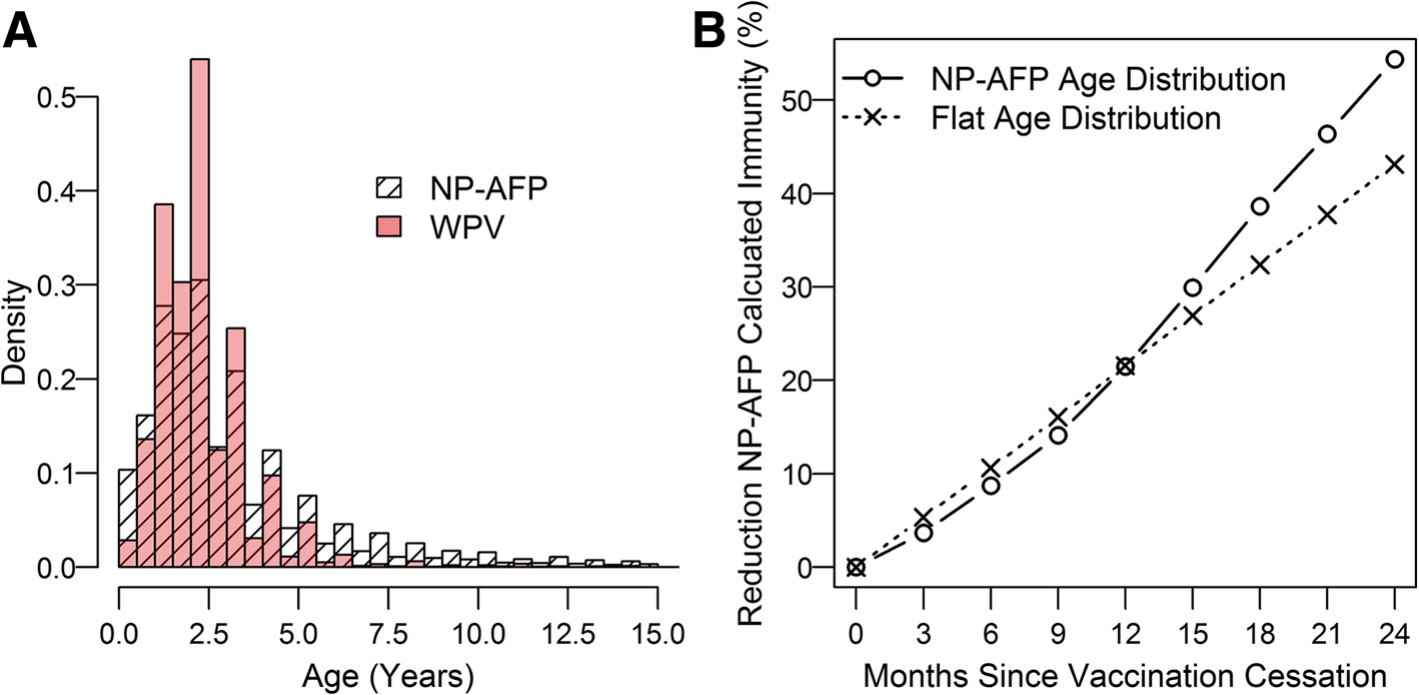
**AFP age distributions and implied impact of vaccination interruptions. (A)**, Comparison of age distribution of WPV-AFP cases and NP-AFP cases between Jan 2004 and June 2013. **(B)**, Reduction in calculated NP-AFP immunity in period of no vaccination based on flat under-five age distribution and NP-AFP under-five age distribution in **(A)**.

The primary source of information used in this analysis is the Nigerian Acute Flaccid Paralysis (AFP) surveillance database (maintained by the Nigerian WHO). When a childhood AFP case is reported, two stool samples are collected and tested for poliovirus. Information regarding age, onset date, parent-reported oral polio vaccine (OPV) dose history, and clinical symptoms is collected as part of the initial case investigation, before the laboratory polio diagnosis is completed. AFP can be caused by viruses other than WPV, though the distribution of these viruses and other causes of AFP are not well understood [27]. AFP cases without confirmed poliovirus in either stool sample, that is, non-polio AFP (NP-AFP), are considered to be a random sample of the population [28-30]. The age distribution of NP-AFP cases is peaked between 18 and 24 months (Figure 1A).

**Figure 2:**
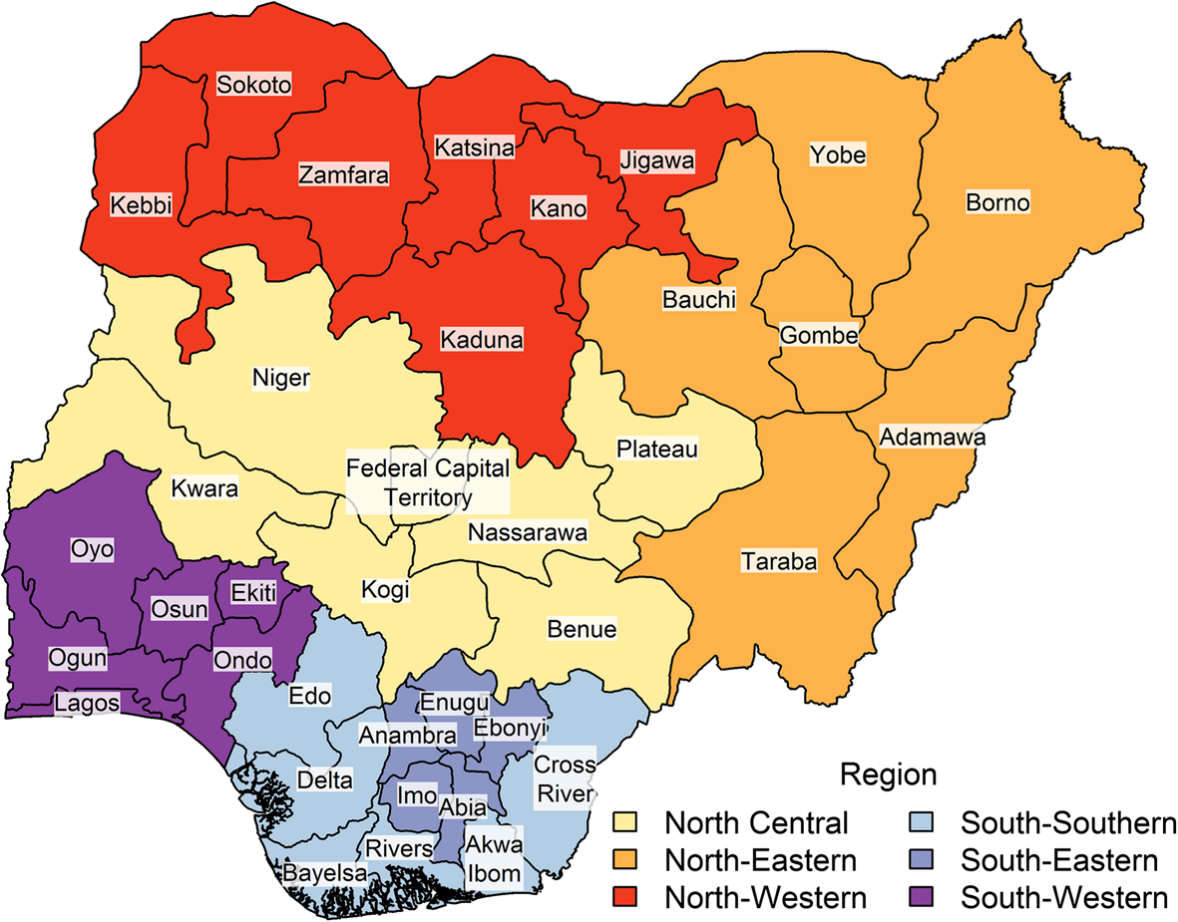
**Map of Nigerian states.** Color signifies geopolitical region.

District (Local Government Area) population estimates were taken from the 2006 Nigerian National Census [31]; absent specific demographic data for each district, the under-five population was estimated to be 20% of the total population of each district^a^. Each district’s population density was calculated by dividing its under-five population by its area computed using the district polygon file [32]. For reference, Figure 2 shows a map of Nigeria.

Individually reported dose histories, vaccine-specific campaign exposures, a probability model for campaign participation, and vaccine-specific OPV efficacies based on AFP surveillance data [33] were used to calculate the probability that an individual is protected against paralysis from each WPV serotype (Additional file 1, Eq. S1) [34]. Individual campaign exposure was defined as the number of SIA campaigns occurring between birth and the date of paralysis onset, based on the historical SIA calendar in the district.

Both polio AFP and NP-AFP cases were linked to districts in Nigeria and binned into six-month periods: December through May or June through November. In the following, the former period is referred to as the first half of a year and the latter as the second half of a year; for example, the first half of 2008 refers to the period from December 2007 through May 2008.

District-level OPV-induced population immunity was taken to be the mean calculated OPV-induced immunity of all NP-AFP cases in children under five years-old occurring in a district within a six-month period. District zero-dose fraction was computed as the proportion of NP-AFP cases for children 0 to 59 months old for which there were reported zero OPV doses in a district within a given six-month period.

Recent caseload is equal to the total number of WPV1 or WPV3 cases in a district in the previous six months. Recent neighboring caseload, also sero-specific, is the total number of WPV1 or WPV3 cases in bordering districts in the previous six months. We used AFP and campaign data from June 2004 through May 2013.

### Statistical methods

**Figure 3:**
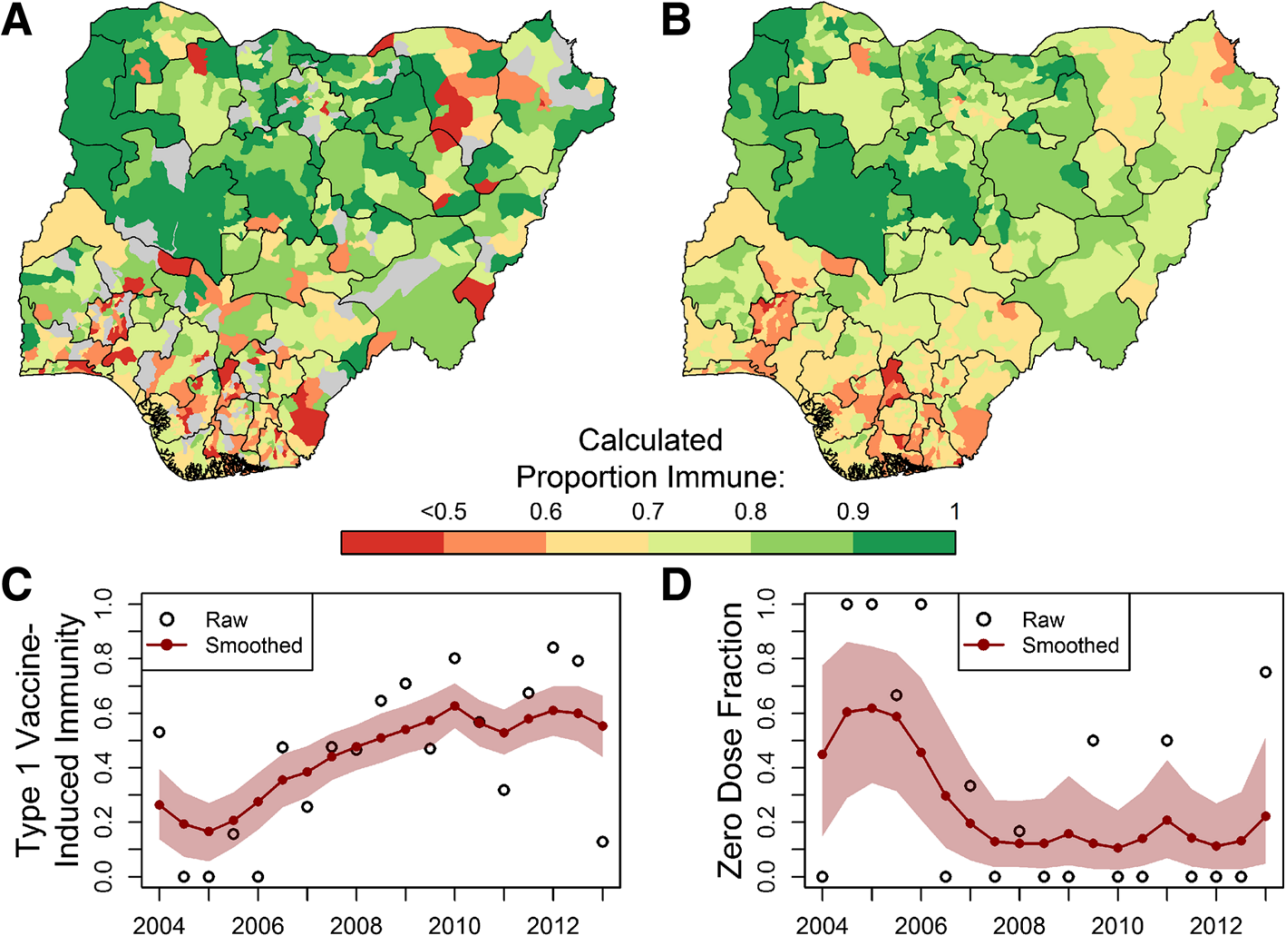
**Impact of statistical smoothing model. (A)**, Map of district NP-AFP WPV1 vaccine-derived immunity, December 2012 through May 2013. Districts colored gray if no data NP-AFP cases reported in this interval. **(B)**, Map of smoothed district WPV1 immunity estimates for the same period. See Discussion for explanation of the relatively low calculated immunity in southern districts. **(C, D)**, Raw versus smoothed indicators over time in Maiduguri, Borno district for WPV1 immunity estimates **(C)** and zero-dose fraction **(D)**. The shaded region represents the point-wise 95% posterior credible interval (CI) of parameter mean estimates.

As only a small number of NP-AFP cases occur within a single district within six months, the empirical estimates of OPV-induced immunity and zero-dose fraction are highly variable from one time period to another: more than 10% of zero-dose fractions in adjacent time periods decreased by more than 0.1 (see Figure 3C, D), an unrealistic difference given the under-five demographics. We adjusted these estimates using a hierarchical temporal smoothing model (see Additional file 1, Eq. S2, S3, S4, S5). Model estimates over time for Maiduguri district in Borno are presented as an example. The posterior mean of the smoothed calculated immunity and zero-dose fraction estimates were then used as indicators in the statistical models of WPV transmission.

WPV1 and WPV3 caseloads were modeled using a Poisson hurdle model: a two-part model consisting of a Bernoulli component that models the probability of reporting one or more WPV cases and a truncated Poisson portion that models the number of WPV cases in infected districts (districts reporting one or more WPV cases; see Additional file 1, Eq. S8, S9, S10) [35,36]. For each component, we included spatially and non-spatially dependent random effects to account for unobserved spatially correlated risk factors [37,38]. Together, these terms enable both local and global borrowing of information, respectively. A bivariate conditional autoregressive (CAR) prior was used to model spatial random effects, and a bivariate normal prior was used to model non-spatial random effects, as we expect areas with greater rates of WPV transmission to report larger case counts [26].

A Bayesian estimation approach was utilized, and models were fit using WinBUGS 1.4.3 [39] and the R2WinBUGS and CODA packages [40,41] in R [42]. Diffuse priors and hyper-priors were used for the regression coefficients and the random effect precisions. The convergence of Markov chain Monte Carlo (MCMC) models was checked through visual inspection of chains and the Gelman-Rubin potential scale reduction factor [43,44]. The selected model was determined using an iterative subtractive approach to arrive at the most parsimonious model with the lowest deviance information criterion (DIC), a well- described method for quantifying the quality of a fit [25,45] (see Additional file 1 for full model details).

The accuracy of model predictions was estimated using prospective sampling: predictions were compared to future data excluded from the initial analysis [46]. The selected model was applied historically using data smoothed over a specified time period; the historical caseload in an upcoming window was predicted using the model coefficients estimated using data from prior time periods and covariates from the most recent time period^b^. The predictive power of the model was assessed using receiver operator characteristic (ROC) analysis using the “pROC” package in R [47]. The empirical area under the ROC curve (AUC) was used to quantify the accuracy of model predictions of infected districts (districts reporting >1 WPV1 case) [48]. As a rule of thumb, an AUC of 0.5 to 0.7 indicates marginal predictive power, an AUC of 0.7 to 0.9 indicates moderate predictive power, and an AUC above 0.9 indicates strong predictive power [49].

Uncertainties in the predicted district caseloads and district ranks were estimated using 1,000 samples from the posterior distribution of parameters. District rank was determined by ordering districts according to predicted WPV1 risk with 1 being most at risk and 774 being least at risk. As districts are prioritized based on rank above a certain threshold, we used samples from the posterior distribution of district ranks to estimate the probability of a district belonging to the *N* districts of highest rank.

## Results

### Influence of data smoothing model

Data smoothing resulted in substantial changes in the estimated regression coefficients; the impact of the smoothing model is highlighted in Figure 3. The smoothing model shrinks extreme district estimates from the first half of 2013 towards the mean (Figure 3A, B). We find the calculated population immunity to be low in southern districts (see Figure 2 for map of Nigeria); see Discussion for possible explanations of this phenomenon. The reduction of rapid fluctuations in values over time is highlighted in Figure 3C and D.

The smoothing effectively captures major trends in the data while reducing the amount of variability between adjacent time points: fewer than 1.5% of changes in the smoothed calculated immunity between adjacent time periods are greater than 0.1 (none are greater than 0.15), compared to 49.7% of changes greater than 0.1 for the empirically calculated immunity. Additionally, fewer than 3% of relative immunity decreases between adjacent time periods were greater than 20% in the smoothed immunity model, while 43% of immunity decreases were greater than 20% for empirical population immunity estimates.

### Factors associated with WPV1 transmission

Regression analysis identified key covariates significantly associated with the presence and number of WPV cases at the district level. Model coefficients and random effect variance components for different models are presented in Table 1. The population density was not significantly associated with the presence or number of WPV1 AFP cases in a district. Zero-dose fraction was negatively associated with only the number of cases in a district in the full model; however, because this association is an artifact of collinearity with population immunity (r=−0.77), we did not include it in the selected model (r=−0.63 for type 3 population immunity and zero-dose fraction). Recent caseloads in a district and in its neighboring districts were positively associated with the presence of cases in a district but not with the number of cases. The estimated OPV-induced population immunity is significantly associated with both the presence and number of WPV1 cases within a district.

**Table 1:** Covariate estimates for models using data through May 2013

**Component**	**Variable**	**Full**	**Selected**	**Null**
Poisson	Intercept	-11.21	-11.51	-12.68
		(-11.88, -10.51)	(-11.83, -11.17)	(-13.03, -12.37)
	Population Immunity	-2.50	-1.80	
		(-3.32, -1.66)	(-2.20, -1.44)	
	Zero-Dose Fraction	-0.72		
		(-1.47, -0.02)		
	Density	0.02		
		(-0.07, 0.11)		
	Sqrt Recent Cases	-0.05		
		(-0.11, 0.02)		
	Sqrt Neighboring Recent Cases	0.02		
		(-0.02, 0.05)		
Bernoulli	Intercept	-0.91	-1.11	-3.60
		(-1.64, -0.16)	(-1.42, -0.80)	(-3.80, -3.43)
	Population Immunity	-4.86	-4.74	
		(-5.69, -4.01)	(-5.27, -4.21)	
	Zero-Dose Fraction	-0.19		
		(-1.12, 0.75)		
	Density	-0.02		
		(-0.11, 0.08)		
	Sqrt Recent Cases	0.19	0.19	
		(0.07, 0.31)	(0.07, 0.32)	
	Sqrt Neighboring Recent Cases	0.17	0.17	
		(0.10, 0.23)	(0.10, 0.23)	
	DIC	9263.6	9266.8	10121.3
	N	13932	13932	13932
Variance	Bernoulli CAR^a^	1.51	1.50	1.48
Components		(1.06, 2.08)	(1.04, 2.04)	(1.08, 1.98)
	Bernoulli Ind^b^	0.10	0.10	0.09
		(0.06, 0.17)	(0.05, 0.18)	(0.05, 0.15)
	Poisson CAR^c^	0.49	0.46	0.67
		(0.24, 0.86)	(0.22, 0.79)	(0.39, 1.05)
	Poisson Ind^d^	0.14	0.13	0.15
		(0.08, 0.23)	(0.07, 0.22)	(0.08, 0.23)
	Covariance CAR^e^	0.10	0.07	0.42
		(-0.23, 0.46)	(-0.23, 0.39)	(0.12, 0.77)
	Covariance Ind^f^	-0.01	-0.01	-0.01
		(-0.05, 0.04)	(-0.07, 0.04)	(-0.06, 0.04)

95% credible intervals (CI) are presented in parentheses underneath corresponding parameter estimates.

^a^Spatial random effects in Bernoulli portion of the model.

^b^Non-spatial random effects in Bernoulli portion of the model.

^c^Spatial random effects in Poisson portion of the model.

^d^Non-spatial random effects in Poisson portion of the model.

^e^Spatial random effect covariance.

^f^Non-spatial random effect covariance.

In models of WPV3 in Nigeria, only the OPV-based population immunity was significantly associated with the number of cases in a district in the selected model, while population immunity and recent cases in neighboring districts were associated with the presence of WPV3 cases in a district (see Additional file 1: Table S2).

A null model without covariates is included in Table 1 as a reference; this model only makes use of population and historical outcome information, through spatial and non-spatial random effects. Interestingly, the random effects variance estimates are only marginally higher in this model than in the selected model, though the covariance between spatial random effects in the Poisson and binomial portions is significantly greater than zero. Fixed effect covariates therefore account for a fairly small proportion of the model variance captured by the random effects alone in the null model. This suggests that the time-varying covariates contain additional information not captured by random effects alone.

**Figure 4:**
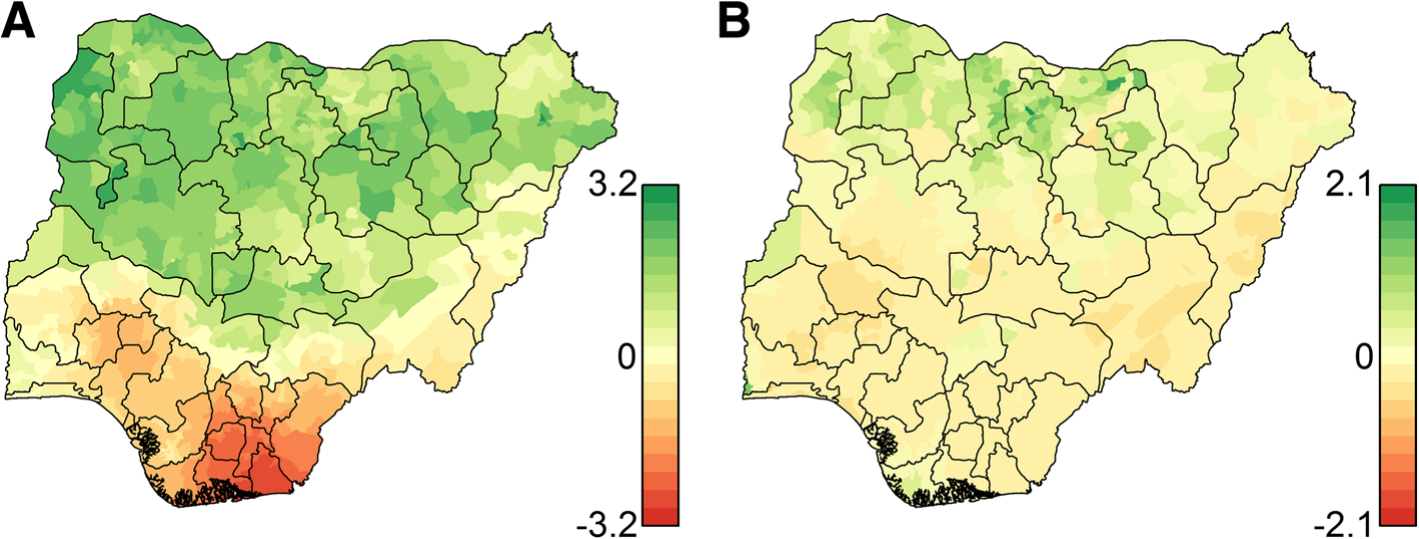
**Magnitude of random effect estimates in the selected WPV1 model.** Estimated with data through May 2013. Sum of posterior mean of spatial and non-spatial random effects in the Bernoulli portion **(A)** and Poisson portion **(B)** of the hurdle model. The estimates are on the logit and log scale, respectively. These figures indicate that the model’s covariates overestimate the risk in southern districts and underestimate the risk in northern Nigeria historically.

The magnitudes of the district-level random effect estimates from the selected model (Table 1) give an indication of where fixed effects alone repeatedly overestimate or underestimate the presence (Figure 4A) or number of cases (Figure 4B) as a result of factors not included in the model that affect WPV1 transmission (see Additional file 1: Figure S1 for similar results for the WPV3 model). Indicators in the binomial portion of the model overestimate the presence of cases in southern districts (possibly due to low estimated OPV-induced population immunity, see Figure 3B) and underestimate the presence of cases in northern districts. Population immunity and population covariates underestimate the number of cases most substantially in districts within Kano and Jigawa states (Figure 4B).

### Predicting WPV1 circulation in districts in Nigeria

**Figure 5:**
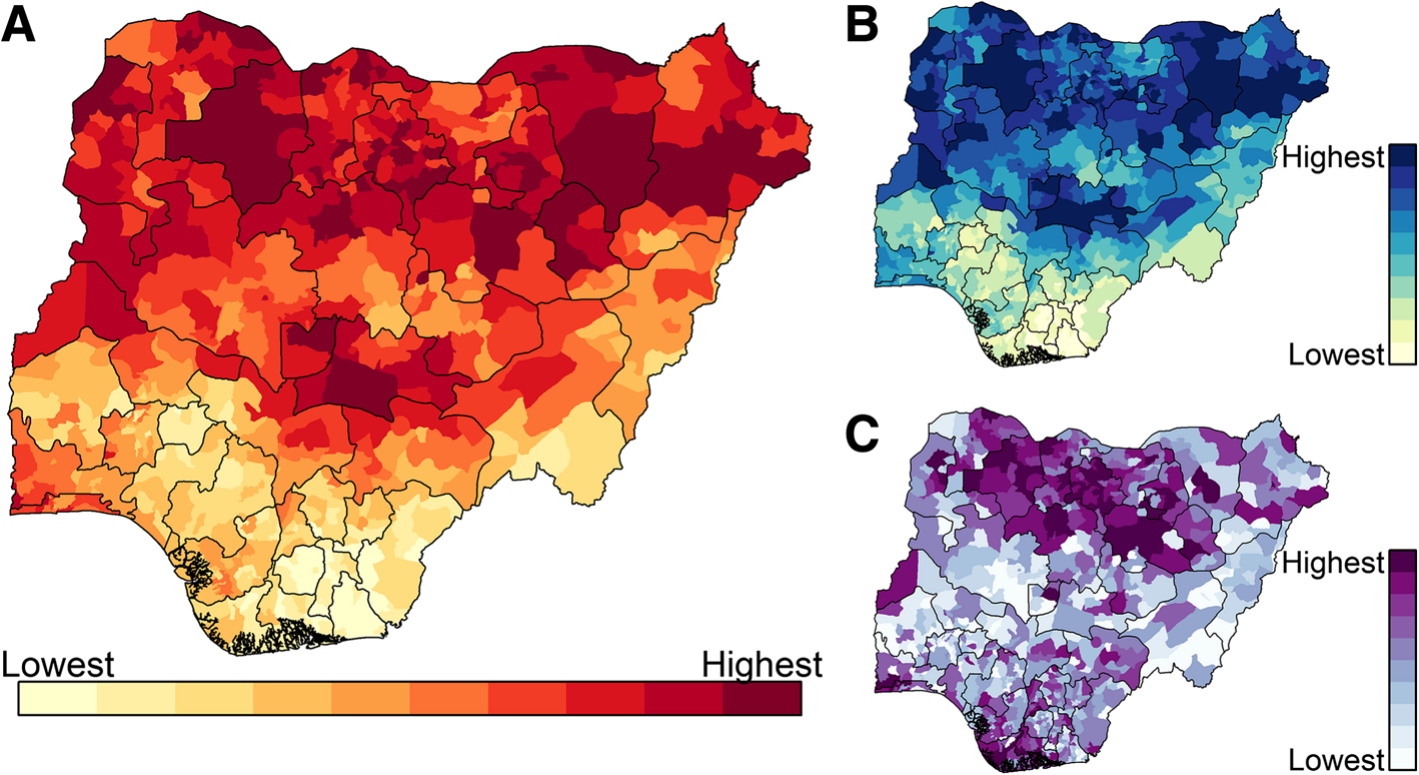
**Model predictions from the selected model for June through November 2013. (A)**, The predicted risk, or expected number of cases, under the model. **(B)**, The predicted probability of one or more reported cases, i.e., the output from the binomial portion of the hurdle model. **(C)**, The predicted number of reported AFP cases given an introduction (one reported AFP case). All estimates based on covariates from December 2012 through May 2013.

Model forecasts are generated by combining predictions from both portions of the model. The spatial distribution of projected risk is largely determined by the binomial portion of the model (Figure 5). The predicted presence and number of cases (outputs of the binomial and Poisson portions, respectively) are distributed differently in space, with the latter clustered in and around Kano state (Figure 5C) and the former highest in the northeast (Borno and Yobe states), northwest (Zamfara state), and north central (Federal Capital Territory and Nasarawa state) regions (Figure 5B).

**Figure 6:**
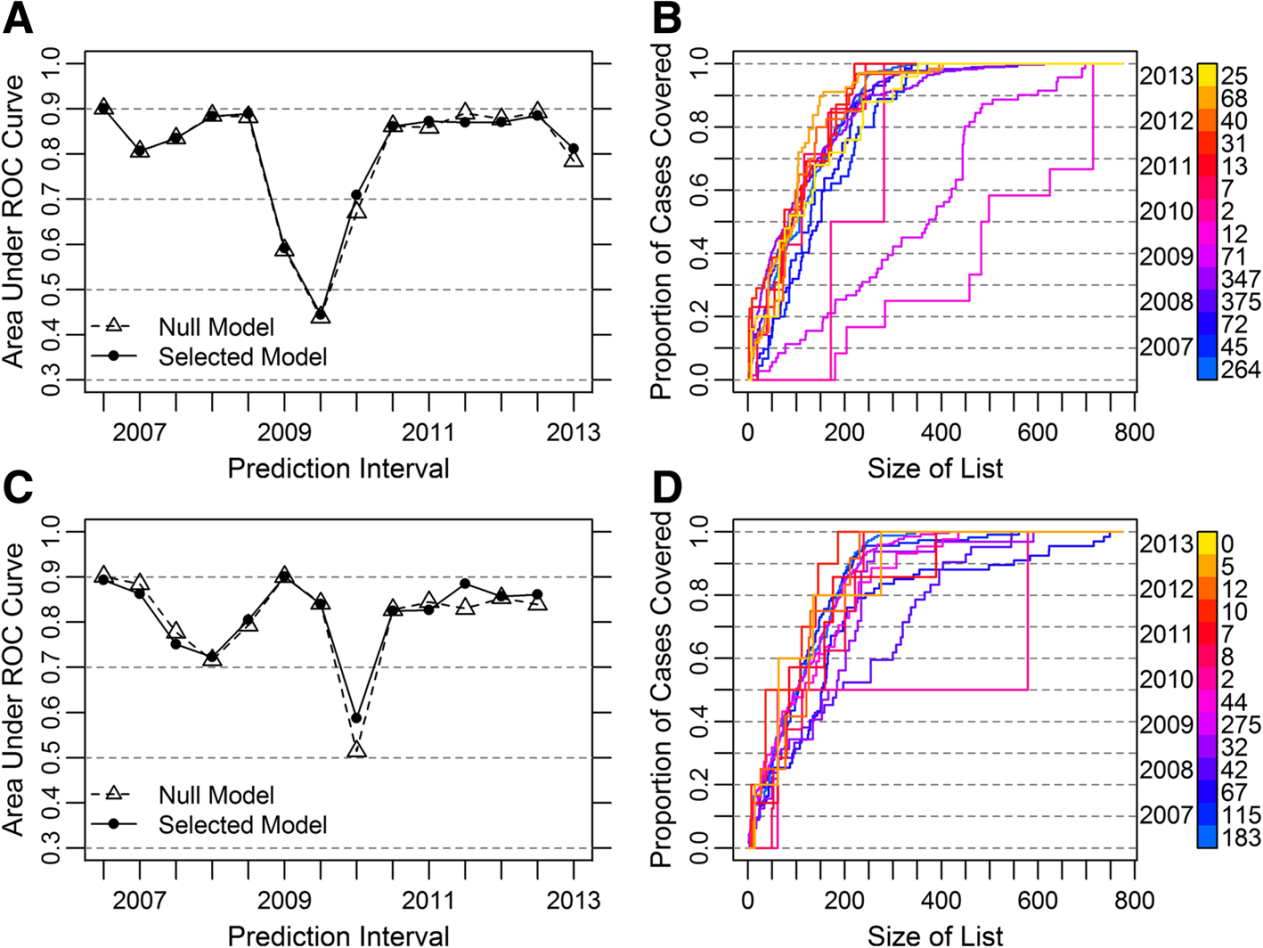
**Forecasting accuracy of historical WPV1 and WPV3 models. (A, C)**, Area under the ROC curve for historical models by prediction interval for WPV1 **(A)** and WPV3 **(C)**. The null model contains only random effects. The selected models include population and estimated population immunity in the Poisson portion and population immunity and recent caseload information in the binomial portion (Table 1 and Additional File 1: Table S2). **(B, D)**, Case sensitivity curves for selected WPV1 **(B)** and WPV3 **(D)** model predictions. The color signifies the prediction interval. The number of cases in the given prediction interval is written to the right of the legend.

To assess the historical predictive power of the model over time, we used the area under the ROC curve (AUC) of a ranked list of districts as a summary statistic of the model’s predictive accuracy (Figure 6A). Within every prediction interval, we estimated both smoothed parameters and model coefficients using the data preceding the interval (Table 2). Over time, the selected model has performed relatively well, with 11 of 14 AUC estimates greater than 0.8 and a mean AUC of 0.86 (SE=0.01) over the past three years. The predictive accuracy was similar for the selected WPV3 model; the mean AUC over the last three years was 0.85 (SE=0.02) (Figure 6C).

**Table 2:** Coefficient estimates from the selected model applied historically

**Component**	**Variable**	**2012**	**2011**	**2010**	**2009**	**2008**
Poisson	Intercept	-11.42	-11.50	-11.60	-11.80	-12.20
		(-11.76, -11.09)	(-11.87, -11.17)	(-11.96, -11.24)	(-12.22, -11.39)	(-12.79, -11.69)
	Population	-1.95	-1.80	-1.60	-1.62	-1.28
	Immunity	(-2.40, -1.51)	(-2.30, -1.31)	(-2.09, -1.10)	(-2.14, -1.11)	(-1.92, -0.61)
Binomial	Intercept	-0.81	-0.54	-0.75	-1.43	-1.89
		(-1.14, -0.46)	(-0.91, -0.17)	(-1.16, -0.35)	(-1.81, -1.02)	(-2.42, -1.36)
	Population	-5.24	-5.69	-5.03	-3.42	-3.17
	Immunity	(-5.82, -4.65)	(-6.34, -5.07)	(-5.71, -4.36)	(-4.18, -2.71)	(-4.19, -2.20)
	Sqrt Recent	0.19	0.18	0.18	0.13	0.18
	Cases	(0.06, 0.32)	(0.05, 0.30)	(0.05, 0.31)	(0.01, 0.26)	(0.04, 0.33)
	Sqrt Neighboring	0.17	0.18	0.16	0.06	0.02
	Recent Cases	(0.10, 0.24)	(0.11, 0.25)	(0.09, 0.23)	(-0.01, 0.13)	(-0.07, 0.10)
	DIC	8732.4	8306.9	8107.3	7798.5	6200.7
	N	12384	10836	9288	7740	6192
Variance	Bernoulli CAR^a^	1.47	1.49	1.41	1.59	1.81
Components		(1.03, 2.03)	(1.02, 2.06)	(0.97, 1.98)	(1.08, 2.23)	(1.26, 2.52)
	Bernoulli Ind^b^	0.11	0.11	0.11	0.11	0.12
		(0.06, 0.19)	(0.06, 0.19)	(0.06, 0.19)	(0.06, 0.19)	(0.06, 0.22)
	Poisson CAR^c^	0.47	0.49	0.50	0.51	0.61
		(0.22, 0.80)	(0.24, 0.85)	(0.24, 0.86)	(0.25, 0.85)	(0.30, 1.02)
	Poisson Ind^d^	0.14	0.14	0.14	0.14	0.18
		(0.07, 0.22)	(0.07, 0.24)	(0.08, 0.23)	(0.08, 0.23)	(0.10, 0.28)
	Covariance CAR^e^	0.04	0.04	0.06	0.26	0.36
		(-0.28, 0.34)	(-0.31, 0.41)	(-0.28, 0.38)	(-0.10, 0.63)	(-0.06, 0.76)
	Covariance Ind^f^	-0.01	-0.01	-0.01	-0.02	-0.03
		(-0.07, 0.04)	(-0.07, 0.04)	(-0.07, 0.04)	(-0.07, 0.04)	(-0.10, 0.04)

Models are estimated using data through the first half of the year that identifies the model. 95% credible intervals (CI) are presented in parentheses underneath corresponding parameter estimates.

^a^Spatial random effects in Bernoulli portion of the model.

^b^Non-spatial random effects in Bernoulli portion of the model.

^c^Spatial random effects in Poisson portion of the model.

^d^Non-spatial random effects in Poisson portion of the model.

^e^Spatial random effect covariance.

^f^Non-spatial random effect covariance.

Interestingly, the selected WPV1 model did not perform better on average than a null model, suggesting that an aggregate measure of spatially smoothed historical risk is equally adept at predicting future infected districts as a model that considers population immunity, recent cases, and recent cases in neighboring districts. The mean AUC of the null WPV1 model over the last three years was 0.86 (SE=0.01). Similarly, the predictive accuracy of the null WPV3 model was comparable to that of the selected WPV3 model; the mean AUC over the last three years was 0.84 (SE=0.02).

In 2009, both the selected and null WPV1 models were very poorly predictive. This result is largely due to a flare-up of WPV1 in southern Nigeria in 2009, in districts that had not previously reported a case (see Additional File 1: Figure S3E, F). The relatively poor predictive power of the model in the first half of 2010 (AUC=0.71) is associated with an uncharacteristically small number of reported WPV1 cases (two cases).As such, a useful quantity for policy makers is the expected proportion of cases contained within a set of the highest risk districts (Figure 6B). Historically in Nigeria, 85 to 90% of future cases have appeared in the 200 highest risk districts, and 55% of future cases have appeared in the 100 highest risk districts under the selected model.

### Uncertainty in prioritization according to predicted caseload

**Figure 7:**
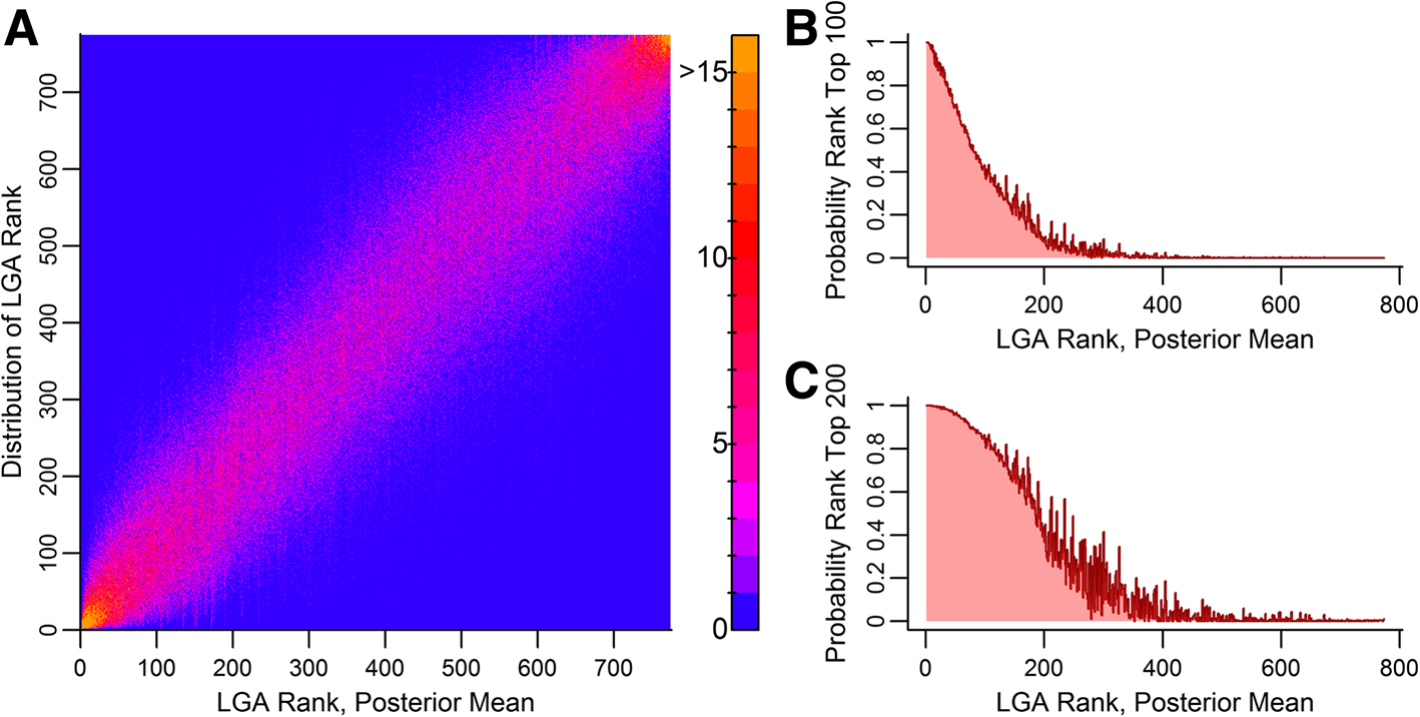
**Model uncertainty in relative district risk ranking.** Prediction interval from 6/2013 through 11/2013. 1,000 posterior samples of model parameters were used to generate 1,000 risk scores and 1,000 ranks for each district. **(A)**, Distribution of posterior district ranks by district. Probability of district rank being in the top 100 **(B)** and in the top 200 **(C)**. Estimates ordered by district rank at posterior mean of parameters.

The model uncertainty can be translated into a district rank uncertainty using 1,000 samples from the posterior distribution of parameters (Figure 7A). The highest and lowest ranked districts exhibit the lowest rank uncertainty. Given a desired list size, the probability of a district being included in the highest risk list can be calculated (Figure 7B, C). Under the selected model, each of the 30 districts with highest predicted risk has more than a 90% probability of being included in the 100 highest ranked districts in June through November 2013.

## Discussion

The smoothing model handles missing data and smooths over unrealistic changes in indicators, such as rapid six-month fluctuations in the under-five immunity estimated from sparse NP-AFP samples. In the absence of any vaccination, the under-five OPV-induced population immunity should decrease by roughly 8-9% in a six-month period (see Figure 1B)^c^. Though thresholds based on this or similar considerations are not explicit in our smoothing model, the smoothed variations between time periods are more realistic than those in empirical data. Smoothing models could be improved by limiting changes between time points to demographically constrained values.

Despite its limitations [50], the OPV-based population immunity, calculated using efficacies derived from case-control population studies [29,33], is strongly associated with the presence and case count of WPV1 at the district level in Nigeria. It is the only covariate significantly associated with the number of cases in a district.

It is expected that the recent caseload is significantly associated only with the expected presence of WPV1, as recent circulation has a mixed effect on the future case count. While a large number of cases within a time period suggests potentially higher transmission (temporally and spatially) from a large infectious reservoir, WPV1 circulation will also boost natural immunity in an area, providing additional protection and reducing the expected caseload in the ensuing time period [50]. Indeed, we find that both recent caseload within a district and in surrounding districts are significantly associated with the probability of a WPV1 case, but not the number of WPV1 cases.

Although increased population density is thought to increase poliovirus transmission potential [51], we find no association in either portion of the model. Due to data limitations, population density was calculated at the district level, which may be a poor representation of the experienced population density on more functional scales of transmission, such as the population density immediately surrounding the household reporting a WPV case. A more fine-grained analysis is needed to better understand the impact of population density of WPV transmission.

The ability to detect an association between zero-dose fraction, an indicator of possible clustering of vulnerability within a district, and the presence and number of WPV1 cases was compromised by a strong correlation between population immunity and zero-dose fraction. The collinearity between these indicators resulted in a negative association between zero-dose fraction and the number of WPV1 cases in a district, while in a model without population immunity, zero-dose fraction has a strong positive association with the number of cases in a district.

Although the historical predictive accuracy is high, covariates in the selected model do not substantially improve the forecasting ability of the model over the null model. In a null model, the random effects capture the mean historical frequency and number of WPV1 cases within a district. This result suggests that although dynamic indicators are associated with WPV1 transmission, historical transmission patterns are stronger predictors of future transmission than available model covariates.

The decrease in performance in 2009 is caused by a flare-up in WPV1 cases in southern districts with no history of WPV1 transmission in our dataset; because the spatial random effects alone are strong predictors of future caseloads, this modeling approach places very little risk in areas with no historical cases in the dataset. The selected model performed as poorly as the null model during this time period, indicating that factors other than calculated OPV-derived population immunity and local transmission contributed to this outbreak.

The outputs from the predictive models are well suited for use in prioritization by public health organizations. In Nigeria, model forecasts are used to inform sub-national SIA planning and allocation of specialized personnel. Prioritization analysis played a critical role in the distribution of technical and administrative field staff—supported by both WHO and the Nigerian government—to the highest risk Local Government Areas across the north during the capacity surge in June 2012. WHO alone grew an initial staff of 744 to over 2,900, an increase of nearly 300%. In addition to supporting SIA planning, monitoring, and evaluation, these personnel have supported household-based micro planning, intensified AFP surveillance activities, and helped strengthen routine immunization activities. Outreach from the federal government to state governors and district chairmen to increase local political buy-in was also heavily increased around this time; prioritizations based on the predictive risk model results were also used to direct this surge in advocacy. These efforts can be partly credited for the absence of cases in 2013 from the northwest of Nigeria, the area of focus for political engagement in the latter half of 2012. F Since June 2013, model outputs have been combined with results and input from the National Primary Health Care Development Agency (NPHCDA), Centers for Disease Control and Prevention (CDC), and WHO to categorize a subset of districts as “high risk” and “highest risk”. This categorization has been used to direct a number of interventions across partner organizations in Nigeria. Management support teams, comprising high level personnel from NPHCDA and other GPEI partner agencies, are deployed to prioritized districts seven to ten days before a SIA campaign to address challenges limiting vaccination coverage. Prioritized districts are selected for advocacy visits targeted at local political, traditional, and religious leaders. Additionally, supplemental logistics funds are provided to prioritized districts to enhance the ability of teams to vaccinate hard-to-reach and scattered settlements. The tracking of vaccinators using GPS-enabled smart phones has been targeted to prioritized districts. Often, these districts receive the first implementation of an intervention planned to eventually deploy across northern Nigeria.

Outside of national program planning, district prioritization categorizations are closely monitored by state governors and district chairmen. Because of additional support, a prioritized district reporting a WPV case is held more accountable than a non-prioritized district.

The quantification of uncertainty in risk rank can also be a useful tool for policy makers. As changes in prioritization are an additional strain on resources (due to required reallocation of people and materials), the quantification of uncertainty in rank can enable objective decisions regarding changes to the prioritization of an area. If a district is newly ranked in the top 100 or 200 highest risk areas, policy makers may only want to prioritize if there is a high level of certainty that the district belongs in the highest risk group.

One aspect not considered in the model was the migration and transport structures connecting non-neighboring districts. With data to inform such structures, we may be better able to predict infection in naive southern districts. WPV1 case information was only available beginning in 2004 for this study. More historical data, with instances of transmission in these areas, could also have improved the forecasting accuracy of the model during these time periods. Seasonal variations and trends, such as those used in predictive models for meningitis in France and Mali [20,21], could be incorporated, though the six-month time scale of model predictions required due to data sparsity may be too large for this technique to be useful.

Additional covariates or more representative data are needed to more fully understand WPV transmission in Nigeria. In the selected model, a substantial amount of residual spatial variation remains, as is demonstrated in Figure 4. This variation may be due to other factors such as poverty, malnutrition, sanitation, and level of health services, which influence WPV transmission potential and population vaccine efficacy [52,53]. In addition, the inclusion of a number of operational factors could greatly improve the model. Indicators capturing district management performance, training quality, vaccinator selection, population accessibility (the presence of hard-to-reach areas within districts, which may be seasonal), and non-compliance are currently missing from the model. Such indicators are an important part of regular program operations. Furthermore, these indicators could be more dynamic than current smoothed indicators, thus improving the responsiveness of model outputs to short-term changes in performance.

Based on the magnitude of district-level random effects, we find that in large portions of northern Nigeria covariates underestimate the probability of the presence of WPV1 in a district, while in southern Nigeria, these covariates overestimate the probability of WPV1 presence. The latter could occur partly because we assume that vaccine efficacy is the same throughout the country, which results in relatively low population immunity estimates in southern districts (Figure 3). This observation is a direct result of the differential number of SIA campaigns executed in northern and southern Nigeria historically: over the last few years, more than twice as many campaigns have been carried out in northern states than in southern states. There is evidence and theory to suggest that vaccine efficacy is not uniform across Nigeria; it may actually be higher in southern Nigeria [33,54], possibly due to a lower burden of non-polio enteroviruses, which are known to interfere with OPV efficacy [55,56]. OPV-derived population immunity estimates based on geographically sensitive vaccine efficacies could resolve this anomaly and strengthen the measured association between calculated population immunity and WPV transmission.

There are several possible sources of statistical bias in our analysis. Though we treated NP-AFP as a random sample of the population in an area, NP-AFP may be under-representative of subpopulations in a district, especially higher risk sub-populations in areas with worse sanitation and less access to health services. This NP-AFP bias is also likely to vary by location. Though the annual rate of NP-AFP (8/100,000 under fifteen) is higher than the minimum WHO guideline (2/100,000 under fifteen), small sample sizes limit the temporal and spatial resolution of this analysis.

Another limitation of this analysis is the recall bias that arises from the oral history collected from the mothers of reported AFP cases. It is possible that they may over- or under-report the number of OPV doses received by the child. It is also possible that the literacy level of the caregivers may influence the accuracy of reported doses. Our analysis does not make allowances for the impact of insecurity on the observed AFP detection rates in some states, such as Borno, Yobe and Kano; the effects may be more meaningful in data collected since December 2012.

There likely exist important heterogeneities in WPV transmission and associated factors below the district level: SIA activities are often planned and carried out at the subdistrict (ward) level in Nigeria. The mean population immunity may be poorly representative of at-risk populations, which will attenuate estimated relationships in the model. Certain populations, such as nomadic peoples [57], may be missed by routine AFP surveillance; in this case, there may be additional WPV cases not included in the analysis. Poorly performing wards may persist in districts with low average risk, as each ward has a focal person responsible for vaccinator selection and training. The population diversity within a district, which often include both rural and urban environments, suggests that the ward level is a more representative level for analysis, although we are severely constrained by a lack of historical data and sparse sample sizes at the subdistrict level.

## Conclusion

The model developed in this study successfully predicts districts at risk for future wild poliovirus cases in Nigeria. Furthermore, the smoothing model handles missing data and smooths over unrealistic changes in important covariates. By quantifying uncertainty in risk ranking, we minimize the likelihood that decision makers respond to stochastic fluctuations in predicted risk over time. Predictive model outputs are well suited for use by public health organizations: in Nigeria, model forecasts are used to inform sub-national SIA planning and to direct diverse interventions.

In order to more fully understand WPV transmission in Nigeria, additional covariates or more representative data are necessary. Heterogeneity in indicators or outcomes below the district level were not considered by this analysis due to data limitations, though there is evidence that this may be important. In addition, better metrics of connectedness between districts—informed by road networks or other transportation structures–could also have improved our model.

In addition to its usefulness for polio eradication through maximizing the impact of political and financial resources, this modeling approach could be extended to other vaccine preventable diseases (VPDs), such as measles. With adequate surveillance data, this type of predictive model provides a principled way of prioritizing administrative areas in future control or elimination efforts.

## Endnotes

^a^ This is compatible with a relatively low life expectancy, especially in northern Nigeria. The 2008 Demographic and Health Survey (DHS) in Nigeria estimates an under-five proportion of 0.171 [58]. In this analysis, the exact fraction is not particularly crucial; as a single value is used for all districts, the relative populations of the districts are preserved.

^b^ For example, to make predictions about the second half of 2008 (June to November 2008), we smoothed indicators through May 2008, and estimated a model with data from July 2004 through May 2008. We then used population immunity and caseload information from the first half of 2008 (December 2007 through May 2008) to make predictions and rank districts according to model outputs.

^c^ Waning of individual immunity has been documented [59,60]; however, this should not be relevant on a six-month time scale.

## Abbreviations

AFP: acute flaccid paralysis; AUC: area under the curve; CDC: Centers for Disease Control and Prevention; CAR: conditional autoregressive; CI: credible interval; DHS: Demographic and Health Survey; DIC: deviance information criterion; GPEI: Global Polio Eradication Initiative; MCMC: markov chain monte carlo; NP-AFP: non-polio acute flaccid paralysis; NPHCDA: National Primary Heath Care Development Agency; OPV: oral polio vaccine; ROC: receiver operator characteristic; SIA: supplementary immunization activity; VPD: vaccine preventable disease; WHO: World Health Organization; WPV: wild poliovirus; WPV1: wild poliovirus type 1; WPV3: wild polio virus type 3.

## Competing interests

The authors declare that they have no competing interests.

## Authors’ contributions

AUB designed the analytical approach, carried out the statistical analysis, and wrote the first draft of the manuscript. HML advised on the analytical approach and helped draft the manuscript. MAP helped conceive of the study and helped draft the manuscript. FS assisted in interpretation of results and helped draft the manuscript. SB assisted in interpretation of results and helped draft the manuscript. HH helped conceive of the study and contributed to the analytical approach. PAE helped conceive of the study and help draft the manuscript. GCC helped conceive of the study, contributed to the analytical approach, and helped draft the manuscript. All authors read and approved the final manuscript.

## Pre-publication history

The pre-publication history for this paper can be accessed here:

http://www.biomedcentral.com/1741-7015/12/92/prepub

## Supplementary Material

Additional file 1 **Supplemental information.** Document with methodological details and additional results and tables referenced in manuscript. Click here for file
