# Supplementary material for: Predictive spatial risk model of poliovirus to aid prioritization and hasten eradication in Nigeria
Source: BMC Med. 2014 Jun 4;12:92. doi: 10.1186/1741-7015-12-92 (PMC4066838; doi:10.1186/1741-7015-12-92)
Supplement: Additional file 1 — Supplemental information. Document with methodological details and additional results and tables referenced in manuscript. [file 1741-7015-12-92-S1.pdf]

Supplemental Information: Predictive spatial risk model of poliovirus to aid prioritization and hasten eradication in Nigeria

Alexander M Upfill-Brown[1*], Hil Lyons[1], Muhammad A Pate[2], Faisal Shuaib[3,4,5], Shahzad Baig[4,6], Hao Hu[1], Philip A Eckhoff[1], Guillaume Chabot-Couture[1]

1 Institute for Disease Modeling, Intellectual Ventures, Bellevue, WA, USA
2 Duke Global Health Institute, Duke University, Durham, NC, USA
3 National Polio Emergency Operations Center, Abuja, Nigeria
4 National Primary Health Care Development Agency, Abuja, Nigeria
5 University of Alabama at Birmingham, Birmingham, AL, USA
6 Kano Polio Emergency Operations Center, Kano, Nigeria
[*] Email: aupfillbrown@intven.com

Population Immunity Model

Type specific campaign exposure is calculated for all NP-AFP individuals with valid dose history and age information. First, the campaign exposure—number of historical campaigns in the district where the NP-AFP case was reported during the interval between the birth-date of the case and the date of onset—is counted. This information is combined with type-specific vaccine efficacies from Table S1. These type-specific efficacies are based on estimates from a case-control model in [1], with additional methodology presented in [2].

In Equation S1, the expected type specific immunity, $E(Im)$, is calculated with type specific efficacies, Eff, and the number of vaccine–specific campaign exposures, n, for tOPV, bOPV, and mOPV: t, b, and m respectively.

$$E(Im) = 1 - \left(\frac{(1 - \text{Eff}_t) \cdot n_t + (1 - \text{Eff}_b) \cdot n_b + (1 - \text{Eff}_m) \cdot n_m}{n_t + n_b + n_m} \right)^{\text{RepDoses}} \tag{S1}$$

District calculated population immunity is taken as the mean of the individual type specific OPV-derived immunity estimates occurring in the district within a six month interval. The empirical district estimates are then smoothed using the method described in the Indicator Smoothing Model section of this manuscript.

We did not re-weight district estimates to make them more representative of the under-five population. As is apparent in Fig. S1A, the age distribution of NP-AFP cases is highly peaked at 12-30 months, roughly similar to the age distribution of confirmed WPV1 cases. Under-five NP-AFP immunity estimates are therefore biased towards 1 and 2 year-olds, the age group with the highest burden of polio AFP.

Based on the age distribution of NP-AFP cases and average NP-AFP immunity by age group since 2011, we would expect a 8-9% drop in estimated NP-AFP immunity in a 6 month period of no vaccination (Fig. S1B). This is less of a reduction than we would expect based on a flat under-five population distribution as NP-AFP is under-representative of 0-6 month-olds. Larger immunity drops are possible based on a different immunity distributions by age.

There are a number of issues with calculating population immunity in this manner. First, we use the same efficacies across Nigeria. Additional work by [1] suggest that vaccine efficacies are actually substantially higher in southern Nigeria than northern Nigeria. Additionally, we do not account for WPV induced population immunity or immunity acquired from the circulation of OPV. Children infected by WPV without developing paralysis are effectively immune against WPV paralysis in the future. Because OPV is a live virus, it is also capable of being transmitted and acquired secondarily by an individual not directly receiving a dose. Because there is little data to guide the estimation of these latter two drivers of population immunity, we limit ourselves here to direct OPV-induced population immunity.

Indicator Smoothing Model

Noisy empirical estimates based on small sample sizes were smoothed using a temporal hierarchical model. For each historical forecasting model, data from preceding time periods were smoothed separately to simulate conditions as if the model was being run in the past.

For OPV-induced immunity, empirical estimates, I, from district j in province i in time interval t were were modeled as normally distributed with mean X_{1ijt} and variance σ^2 inversely proportional to the number of NP-AFP cases reported, n_{1ijt} (Eq S2). Latent variable X_{1ijt} is a function of random effects for mean province immunity, first-order random walk for province immunity at time t, P_{1it}, mean district immunity, and a first-order random walk for district immunity at time t, D_{1ijt} (Eq S3). Because X_{1ijt} is not naturally limited between 1 and 0, any posterior values outside this range were truncated. If there were no NP-AFP cases in district j in interval t, n_{ijt} was set to one.

$$I_{ijt} \sim \mathrm{N}\left(X_{ijt}, \frac{\sigma^2}{n_{ijt}}\right) \tag{S2}$$

$$X_{ijt} = \alpha + P_{1i} + P_{1it} + D_{1ij} + D_{1ijt} \tag{S3}$$

For zero-dose fraction, the number of zero-dose NP-AFP cases, NZ, from district j in province i in time interval t were modeled as binomially distributed with success probability Y_{1ijt} and number of trials equal to the number of NP-AFP cases reported, n_{1ijt} (Eq S4). The logit of the smoothed zero-dose fraction, Y_{1ijt}, is a function of district and province mean and temporally smoothed random effects, similar to the immunity calculation above (Eq S5).

$$NZ_{ijt} \sim \mathrm{Binom}(Y_{ijt}, n_{ijt}) \tag{S4}$$

$$\mathrm{logit}(Y_{ijt}) = \beta + P_{2i} + P_{2it} + D_{2ij} + D_{2ijt} \tag{S5}$$

An inverse diffuse gamma distribution was used for the variance components of the non-temporal (Eq S6) and temporal random effects (Eq S7). The identical prior structure as outlined below was used for district-level random effects terms in the smoothing model. The first order random walk prior on the temporal smoothing terms, P_{nit} and D_{nit} where $n = 1$ or 2, is summarized below (Eq S7). c represents the adjacency matrix and takes a value of 1 if t and k are proximal time periods and 0 if they are not adjacent in time.

$$P_{ni} \sim \mathrm{N}(0, \sigma^2_{P_n}) \tag{S6}$$

$$P_{nit} | P_{nit \neq k} \sim \mathrm{N}\left(\frac{\sum_{k \neq t} c_{tk} P_{nik}}{\sum_{k \neq t} c_{tk}}, \frac{\sigma^2_{P_{nt}}}{\sum_{k \neq t} c_{tk}}\right) \tag{S7}$$

The smoothed values of type specific calculated immunity, X_{ijt}, and zero-dose fraction, Y_{ijt}, were then used as indicators in statistical models for WPV transmission. Separate models were estimated for WPV1 and WPV3. Results from WPV3 specific analysis are summarized below.

WPV Transmission Model

WPV1 and WPV3 caseload was modeled with a Poisson hurdle model (Eq S8): a two-part model consisting of a Bernoulli component (Eq S10) that models the probability of reporting one or more WPV cases and truncated Poisson portion (Eq S9) modeling the number of WPV cases in infected districts [3,4]. In

the equations below, j indexes district and t indicates time interval. Y_{jt} represents the case count. β represents a vector of fixed effects and X_{jt} represents a vector of corresponding covariates. Each portion of the model contains spatial, u_{1j} and u_{2j}, and non-spatial, v_{1j} and v_{2j}, random effects, which are fixed through time. District population, Pop_j, was included as an offset in the Poisson portion as expected counts are based on a rate per population.

$$E(Y_{jt}) = p_{jt} \cdot \frac{\lambda_{jt}}{1 - e^{-\lambda_{jt}}} \tag{S8}$$

$$\log(\lambda_{jt}) = \alpha_0 + \log(Pop_j) + \boldsymbol{\beta_1}\mathbf{X_{1jt}} + u_{1j} + v_{1j} \tag{S9}$$

$$\text{logit}(p_{jt}) = \beta_0 + \boldsymbol{\beta_2}\mathbf{X_{2jt}} + u_{2j} + v_{2j} \tag{S10}$$

The spatial and non-spatial random effects in the Poisson hurdle model of WPV transmission (Eq S8–S10) were modeled using a bivariate normal prior to allow for the borrowing of information between the Poisson and Bernoulli portions of the model.

$$\begin{aligned} &\text{for } j = 1, \ldots, 774 \\ &\begin{bmatrix} v_{1j} \\ v_{2j} \end{bmatrix} \sim \text{MVN}\left(\begin{bmatrix} 0 \\ 0 \end{bmatrix}, \begin{bmatrix} \sigma^2_{1\text{Ind}} & \sigma^2_{12\text{Ind}} \\ \sigma^2_{12\text{Ind}} & \sigma^2_{2\text{Ind}} \end{bmatrix} \right), \end{aligned} \tag{S11}$$

$$\begin{bmatrix} u_{1j} | u_{1j \neq k} \\ u_{2j} | u_{2j \neq k} \end{bmatrix} \sim \text{MVN}\left(\begin{bmatrix} \frac{\sum_{k \neq j} c_{jk} u_{1k}}{\sum_{k \neq j} c_{jk}} \\ \frac{\sum_{k \neq j} c_{jk} u_{2k}}{\sum_{k \neq j} c_{jk}} \end{bmatrix}, \begin{bmatrix} \frac{\sigma^2_{1\text{CAR}}}{\sum_{k \neq j} c_{jk}} & \frac{\sigma^2_{12\text{CAR}}}{\sum_{k \neq j} c_{jk}} \\ \frac{\sigma^2_{12\text{CAR}}}{\sum_{k \neq j} c_{jk}} & \frac{\sigma^2_{2\text{CAR}}}{\sum_{k \neq j} c_{jk}} \end{bmatrix} \right) \tag{S12}$$

The subscript 1 in the equations above corresponds to the Poisson portion of the model, while 2 corresponds to the Bernoulli portion. c is the adjacency matrix, calculated using the district shapefile; c_{jk} takes a value of 1 if district j and k share a border, and c_{jk} equals zero if they do not. MVN signifies the multivariate normal distribution, in this case, of two dimensions.

A diffuse normal prior—mean of zero, variance of 10000—was used for model intercepts and fixed effects. And inverse Wishart distribution prior—second order identity matrix as scale matrix, 2 degrees of freedom—was used for the covariance structure for both spatial and non-spatial random effects. 1,000 posteriors were sampled from 80,000 iterations, with the first 20,000 discarded as a burn-in.

The Poisson hurdle likelihood was used was based on the model presented in [5].

WPV3 Analysis Results

Similar spatial hierarchical models to those estimated using WPV1 data were estimated for WPV3 in Nigeria. The historical forecast accuracy of the model was also assessed using ROC analysis. At the time of writing, WPV3 had not been detected in Nigeria since November 10, 2012. A spatial Poisson hurdle model was applied to historical WPV3 caseload and the selected model was determined by comparing model DIC estimates and the plausibility of estimated associations (Table S2).

In the selected model, calculated population immunity was significantly associated with both the presence and number of WPV3 cases in a district. Like WPV1 models, OPV zero-dose fraction was negatively associated with the number of cases in the full WPV3 model, a result of collinearity with population immunity (see next section), and not included in the selected model. Only the number of

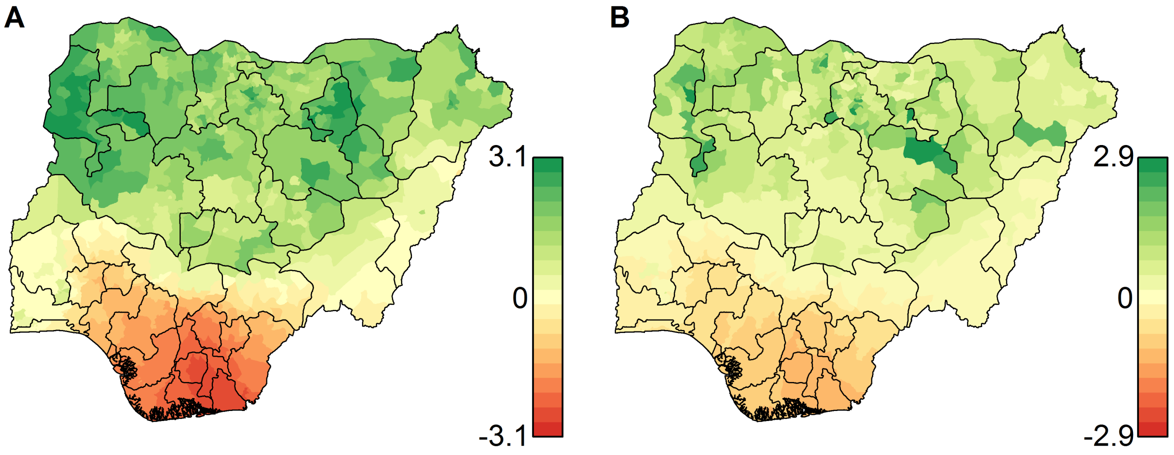

Figure S1. Magnitude of random effect estimates in the selected WPV3 model. Model based on data through May 2013. Sum of posterior mean of spatial and non-spatial random effects in the Bernoulli portion **(A)** and Poisson portion **(B)** of the hurdle model. The estimates are on the log and logit scale, respectively. This figures indicate the model fixed effects are over estimating risk in southern districts and underestimating risk in northern Nigeria.

WPV3 cases in neighboring districts in the previous time period is associated with the likelihood of reporting a WPV3 case. The full and null models are included in Table S2 as a reference.

The random effect magnitude for each district in the selected model is plotted in Figure S2 for the Bernoulli portion and the Poisson portion separately. Fixed effects alone overestimate WPV3 case presence probability and count in the southern Nigeria, especially in Akwa Ibom and Rivers provinces. The number of WPV3 cases given an introduction, Bernoulli portion output, is substantially underestimated by fixed effects alone in only a handful of districts.

The selected model was applied historically with data and model estimates from a given time interval to forecast WPV3 cases in the following six month time interval. Empirical AUC was used to quantify forecast accuracy. Predictive ability is relatively stable over time, with 10/13 of forecast AUCs above 0.80. The mean AUC over the past three years is 0.84. Sensitivity to cases versus list size, another possible metric of forecast accuracy was also employed. Over the past 3 years, the models had predicted 83% of cases in the top 200 highest risk districts and 50% of cases in the top 100 highest risk districts.

Calculated Immunity and Zero-Dose Fraction

In both WPV1 and WPV3 regression models, smoothed zero-dose fraction is positively associated with the number of cases in an area when controlling for type-specific calculated population immunity. Zero-dose fraction was not included in the selected models used for prediction because this result is unintelligible and, furthermore, the result of multicollinearity (Fig. S4). The correlation between population immunity and zero-dose fraction compromises the validity of individual predictor estimates when both indicators are included in the same model. In a model without the population immunity, zero dose fraction is positively association with the number of cases in a district. However, when population immunity is included in the model, the association between zero-dose fraction and immunity is reversed and magnitude is greatly reduced.

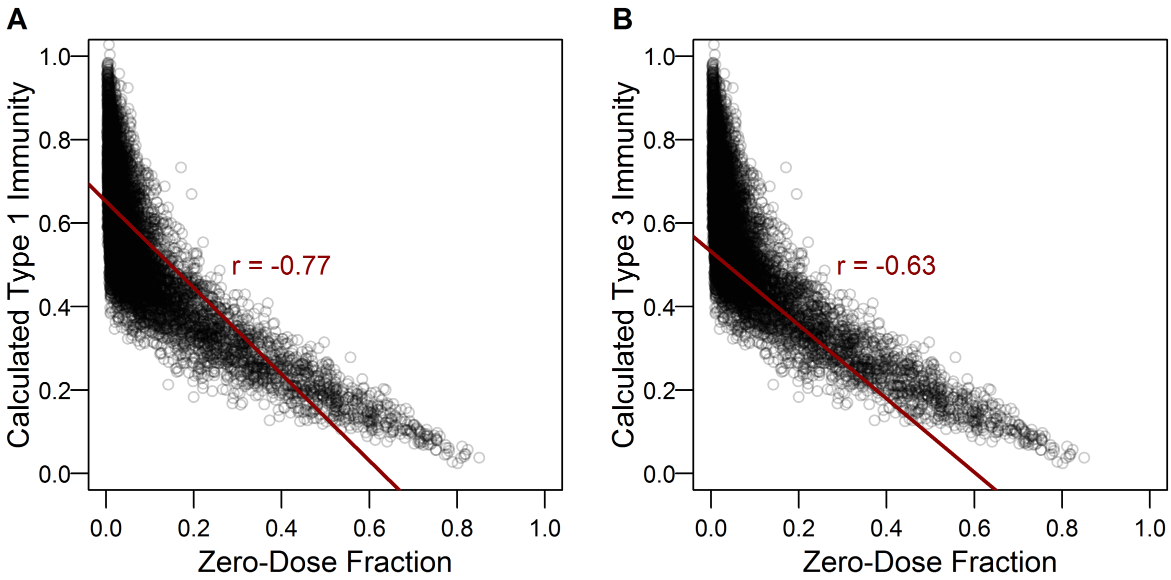

Figure S2. Correlation between zero-dose fraction and population immunity. Correlation between smoothed zero-dose fraction and calculated population immunity in six-month intervals at district level for **(A)** type 1 immunity and **(B)** type 3 immunity. The correlation between type 1 immunity and zero-dose fraction (-0.77) is greater than that between type 3 immunity and zero-dose fraction (-0.63).

WPV1 Infected Districts Over Time

In Figure S5, WPV1 infected districts are plotted over time. The failure of the model forecasts in Figure 4 in 2009 and 2009.5 correspond to time periods where a high relative proportion of cases occurred in southern districts (Fig. S5E, F). Our model was not able to identify risk in these areas using population immunity, recent caseload, and residual historical risk alone. As our model only considers connections between neighboring LGAs, a model defining longer distance relationship might be able to predict outbreaks in southern LGAs as well as transmission across northern Nigeria. District connectivity through road networks could be used to inform such a model.

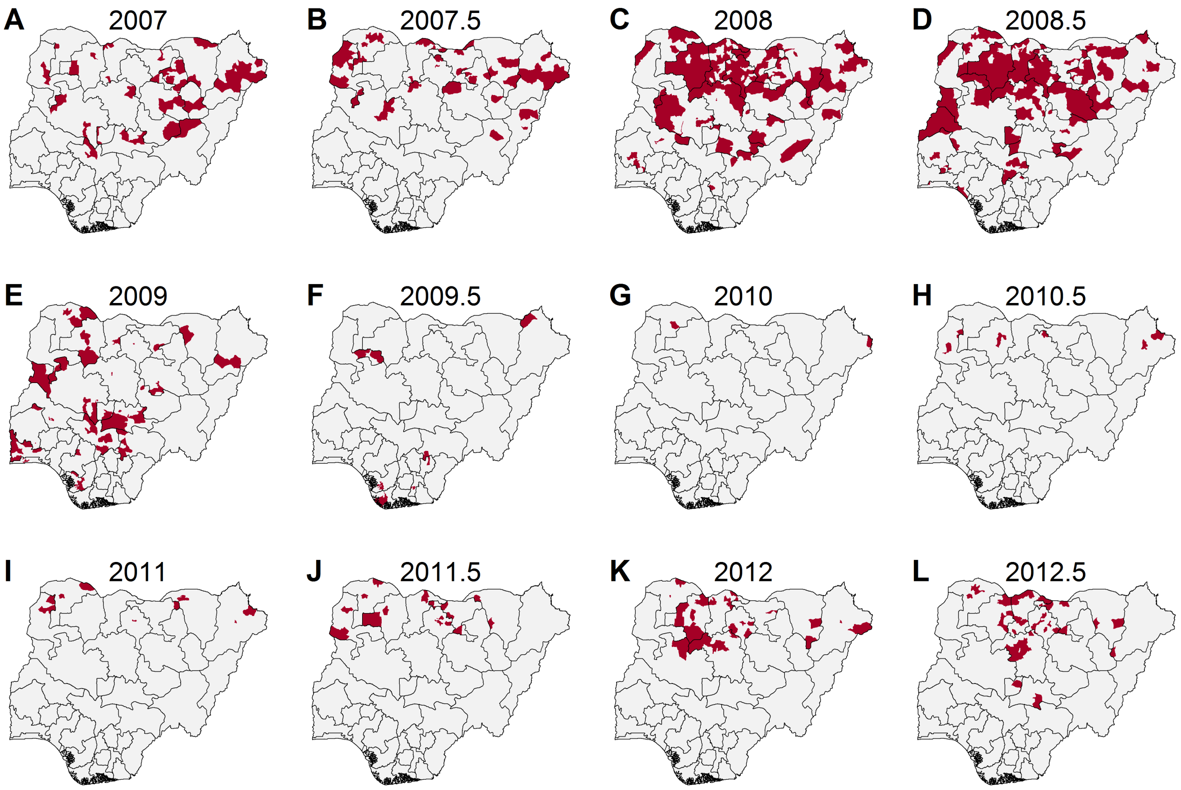

Figure S3. Map of WPV1 infected districts over time by time six-month interval. Infected districts are plotted in red.

References

1. Mangal TD, Aylward RB, Mwanza M, Gasasira A, Abanida E, et al. (2014) Key issues in the persistence of poliomyelitis in Nigeria: a case-control study. Lancet Glob Health 2: e90-e97.
2. Jenkins HE, Aylward RB, Gasasira A, Donnelly CA, Abanida EA, et al. (2008) Effectiveness of Immunization Against Paralytic Poliomyelitis in Nigeria. N Engl J Med 359: 1666–74.
3. Mullahy J (1986) Specification and testing of some modified count data models. J Econom 33: 341–365.
4. Pohlmeier W, Ulrich V (1995) An Econometric Model of the Two-Part Decisionmaking Process in the Demand for Health Care. J Hum Resour 30: 339–361.
5. Neelon B, Ghosh P, Loebs PF (2013) A Spatial Poisson Hurdle Model for Exploring Geographic Variation in Emergency Department Visits. J R Stat Soc Ser A 176: 389–413.

Tables

Table S1. Vaccine efficacies used to calculate NP-AFP case OPV-derived immunity.

	tOPV[a]	bOPV[b]	mOPV[c]
Type 1	19.4	29.5	32.1
Type 2	48.5	-	-
Type 3	18.0	23.8	43.2

[a]Trivalent OPV
[b]Bivalent OPV
[c]Monovalent OPV

Table S2. Covariate estimates for WPV3 models using data through May 2013. 95% credible interval (CI) in parentheses underneath corresponding parameter estimate.

Component	Variable	Full	Selected	Null
Poisson	Intercept	-11.89	-13.03	-13.91
		(-13.00, -10.84)	(-13.73, -12.35)	(-14.62, -13.30)
	Population	-3.76	-1.97	
	Immunity	(-5.49, -2.15)	(-3.06, -0.82)	
	Zero-Dose	-1.27		
	Fraction	(-2.08, -0.46)		
	Denstiy	-0.06		
		(-0.21, 0.07)		
	Sqrt Recent	0.06		
	Cases	(-0.07, 0.19)		
	Sqrt Neighboring	-0.03		
	Recent Cases	(-0.12, 0.07)		
Bernoulli	Intercept	-1.63	-1.69	-4.32
		(-2.43, -0.85)	(-2.07, -1.34)	(-4.58, -4.10)
	Population	-6.72	-6.39	
	Immunity	(-7.70, -5.80)	(-7.13, -5.56)	
	Zero-Dose	-0.35		
	Fraction	(-1.09, 0.36)		
	Density	0.02		
		(-0.09, 0.14)		
	Sqrt Recent	0.05		
	Cases	(-0.13, 0.22)		
	Sqrt Neighboring	0.08	0.10	
	Recent Cases	(-0.01, 0.19)	(0.01, 0.20)	
	DIC	5673.3	5694.3	6197.9
	N	13932	13932	13932
Variance	Bern CAR[a]	1.63	1.66	1.53
Components		(1.09, 2.31)	(1.11, 2.36)	(1.10, 2.07)
	Bern Ind[b]	0.12	0.12	0.10
		(0.06, 0.21)	(0.06, 0.23)	(0.05, 0.18)
	Poisson CAR[c]	0.72	0.73	0.80
		(0.25, 1.42)	(0.29, 1.42)	(0.32, 1.55)
	Poisson Ind[d]	0.39	0.40	0.39
		(0.19, 0.64)	(0.22, 0.63)	(0.20, 0.65)
	Covariance CAR[e]	0.66	0.71	0.76
		(0.12, 1.21)	(0.16, 1.25)	(0.34, 1.27)
	Covariance Ind[f]	-0.05	-0.05	-0.04
		(-0.16, 0.05)	(-0.16, 0.05)	(-0.14, 0.05)

[a]Spatial random effects in Bernoulli portion of the model
[b]Non-spatial random effects in Bernoulli portion of the model
[c]Spatial random effects in Poisson portion of the model
[d]Non-spatial random effects in Poisson portion of the model
[e]Spatial random effect covariance
[f]Non-spatial random effect covariance
